# Detection and Identification of *Acanthamoeba* and Other Nonviral Causes of Infectious Keratitis in Corneal Scrapings by Real-Time PCR and Next-Generation Sequencing-Based 16S-18S Gene Analysis

**DOI:** 10.1128/JCM.02224-20

**Published:** 2021-01-21

**Authors:** Dennis Back Holmgaard, Celine Barnadas, Seyed Hossein Mirbarati, Lee O’Brien Andersen, Henrik Vedel Nielsen, Christen Rune Stensvold

**Affiliations:** a Department of Clinical Microbiology, Slagelse Hospital, Slagelse, Denmark; b European Programme for Public Health Microbiology Training (EUPHEM), European Centre for Disease Prevention and Control (ECDC), Stockholm, Sweden; c Statens Serum Institut, Copenhagen, Denmark; d Institute for Biotechnology and Biomedicine, Technical University of Denmark, Kongens Lyngby, Denmark; e Department of Bacteria, Parasites, and Fungi, Statens Serum Institut, Copenhagen, Denmark; Mayo Clinic

**Keywords:** Denmark, keratitis, NGS, ocular disease, microbiome, parasite infection

## Abstract

*Acanthamoeba* is a free-living amoeba of extensive genetic diversity. It may cause infectious keratitis (IK), which can also be caused by bacteria, fungi, and viruses. High diagnostic sensitivity is essential to establish an early diagnosis of *Acanthamoeba*-associated keratitis. Here, we investigated the applicability of next-generation sequencing (NGS)-based ribosomal gene detection and differentiation (16S-18S) compared with specific real-time PCR for the detection of *Acanthamoeba*. Two hundred DNAs extracted from corneal scrapings and screened by *Acanthamoeba*-specific real-time PCR were analyzed using an in-house 16S-18S NGS assay. Of these, 24 were positive by specific real-time PCR, of which 21 were positive by the NGS assay. Compared with real-time PCR; the specificity and sensitivity of the NGS assay were 100% and 88%, respectively. Genotypes identified by the NGS assay included T4 (*n* = 19) and T6 (*n* = 2). Fungal and bacterial species of potential clinical relevance were identified in 31 of the samples negative for *Acanthamoeba*, exemplified by Pseudomonas aeruginosa (*n* = 11), *Moraxella* spp. (*n* = 6), Staphylococcus aureus (*n* = 2), *Fusarium* spp. (*n* = 4), and Candida albicans (*n* = 1). In conclusion, the 16S-18S assay was slightly less sensitive than real-time PCR in detecting *Acanthamoeba*-specific DNA in corneal scrapings. Robust information on genotypes was provided by the NGS assay, and other pathogens of potential clinical relevance were identified in 16% of the samples negative for *Acanthamoeba*. NGS-based detection of ribosomal genes in corneal scrapings could be an efficient screening method for detecting nonviral causes of IK, including *Acanthamoeba*.

## Abstract (precis)

*Acanthamoeba* is a free-living amoeba of extensive genetic diversity. It may cause infectious keratitis (IK), which can also be caused by bacteria, fungi, and viruses.

## INTRODUCTION

*Acanthamoeba* is a free-living amoeba found primarily in soil and water. The genus exhibits a high degree of genetic diversity, and to date, 20 genotypes have been identified (1). *Acanthamoeba* can cause infectious keratitis (IK), which can lead to blindness if left untreated (2). The overall disease burden of *Acanthamoeba-*associated keratitis (AK) remains low, with an estimated prevalence of 1 to 9 per 100,000 according to the Orphanet database (2). However, the incidence has increased dramatically since the first cases of AK were reported in the 1970s (3). This increase is most likely associated with increased clinical awareness, the development of sensitive tests, and an increase in the number of individuals exposed to risk factors.

*Acanthamoeba*-associated keratitis is often seen in contact lens wearers (4, 5). A recent study from Denmark, which included PCR-based diagnostic data on corneal scrapings tested at our laboratory, revealed that 89% of AK patients were contact lens users, and 49% and 54% had received corticosteroids and treatment for herpes, respectively, before the diagnosis was established (6). In Denmark, an *Acanthamoeba-*positive test result is typically seen in unilateral cases of IK not responding to treatment with chloramphenicol, ciprofloxacin, and/or tobramycin (https://en.ssi.dk/news/epi-news/2016/no-44---2016).

In general, the incidence ranges from 17 to 70 cases per million individuals wearing contact lenses (3). Other events, such as corneal surgery, trauma, or exposure to contaminated water, have also been associated with AK.

An early diagnosis of AK is critical to ensuring a good prognosis (7). However, since IK can also be caused by bacteria, fungi, and viruses with a significant clinical overlap, establishing a diagnosis requires clinical expertise supported by specialized microbiological diagnostics, e.g., real-time PCR (RT-PCR), confocal microscopy, and/or culture using specialized media (8). This was illustrated in a German multicenter study in which 172 cases of AK were reported. Only 23% were initially diagnosed as AK. Several other causes were suggested: 48% were first attributed to herpes simplex virus, 25% were thought to be of bacterial origin, and 3% were wrongly attributed to fungi (9) While an early diagnosis is critical to the prognosis of AK, an average of 2.8 ± 4.0 months (range, 0 to 23 months) of delay between the onset of symptoms and the diagnosis has been reported (9).

Since IK can be caused by a variety of microorganisms and often reflects a polymicrobial coinfection including fungi and/or bacteria in addition to *Acanthamoeba* (10, 11), a strategy to address this situation could be to apply next-generation sequencing (NGS) of ribosomal genes in corneal scrapings in order to screen for all nonviral causes of IK in the early phase of the disease.

In this study, we used a recently developed amplicon-based sequencing assay targeting parasites, fungi, and bacteria based on analysis of nuclear ribosomal genes (16S and 18S rRNA) amplified from genomic DNA extracted directly from clinical corneal scrapings (12, 13). We compared the results obtained by a well-established real-time PCR for *Acanthamoeba* with the detection and differentiation of nuclear ribosomal genes present in corneal scrapings from patients with keratitis in order to (i) evaluate the sensitivity of the 16S-18S assay compared with real-time PCR, (ii) evaluate the robustness of genotyping based on 16S-18S analysis, and (iii) explore the potential of comprehensive 16S-18S analysis in terms of simultaneous detection of clinically relevant fungi, parasites, and bacteria as causative agents of keratitis.

## MATERIALS AND METHODS

### Sampling and reference data.

A total of 200 corneal scrapings collected in 2015 and 2016 were included in this study. These samples, from patients with suspected IK, had been referred for *Acanthamoeba* PCR at the Laboratory of Parasitology at the Statens Serum Institut, Copenhagen, Denmark. Clinicians are advised to take representative and sufficiently large samples in sterile saline water. Samples are usually processed within 24 h of sampling. DNAs had been extracted using the QIAamp DNA blood and tissue kit (Qiagen). The TaqMan-based real-time PCR assay for *Acanthamoeba* spp. in use in our routine diagnostic laboratory was a single-assay modification of the triplex assay previously described by Qvarnström et al. in 2006 (14). All samples had been tested in duplicate by real-time PCR. One water sample (nontemplate; used to test for contamination, including between samples) was included and “DNA-extracted” in each run (12 samples were extracted per run); these water samples were also included in the real-time PCR and NGS assays. Moreover, a water sample is included in the real-time PCR and in the NGS assay. In the real-time PCR, DNA from an *Acanthamoeba* culture (kindly provided by Marianne Lebbad, Public Health Agency of Sweden, Solna, Sweden) was included as a positive control. No positive controls were included in the NGS assay, since this is a microbe profiling assay and was not developed as a diagnostic assay. Both real-time PCR-positive (*n* = 24) and -negative (*n* = 176) samples were included in this study for validation of the 16S-18S assay. Two of the PCR-positive samples were weakly positive, which means that only one of the two duplicates yielded a signal, and this signal reflected a threshold cycle (*C_T_*) value of 40 or more.

### 16S-18S assay: NGS-based detection and differentiation of nuclear ribosomal genes.

Amplification of nuclear ribosomal genes was performed as described previously (13, 15, 16). Briefly, the three following primer sets were chosen for 18S rRNA genes: G3F1/G3R1 (GCCAGCAGCCGCGGTAATTC/ACATTCTTGGCAAATGCTTTCGCAG), G4F3/G4R3 (CAGCCGCGGTAATTCCAGCTC/GGTGGTGCCCTTCCGTCAAT), and G6F1/G6R1 (TGGAGGGCAAGTCTGGTGCC/ACGGTATCTGATCGTCTTCGATCCC). G3 and G6 primers target the V3-V4 hypervariable regions, and G4 targets the V3-V5 hypervariable region of the 18S rRNA gene. For 16S rRNA gene amplification, we used a modified version of the published universal prokaryotic primer set 341F/806R, targeting the V3-V4 hypervariable regions (17). The forward primer, 341F3, had three additional nucleotides attached in the 5′ end (ACTCCTAYGGGRBGCASCAG), and the reverse primer, 806R5, had five additional nucleotides attached in the 5′ end (AGCGTGGACTACNNGGGTATCTAAT).

**FIG 1:**
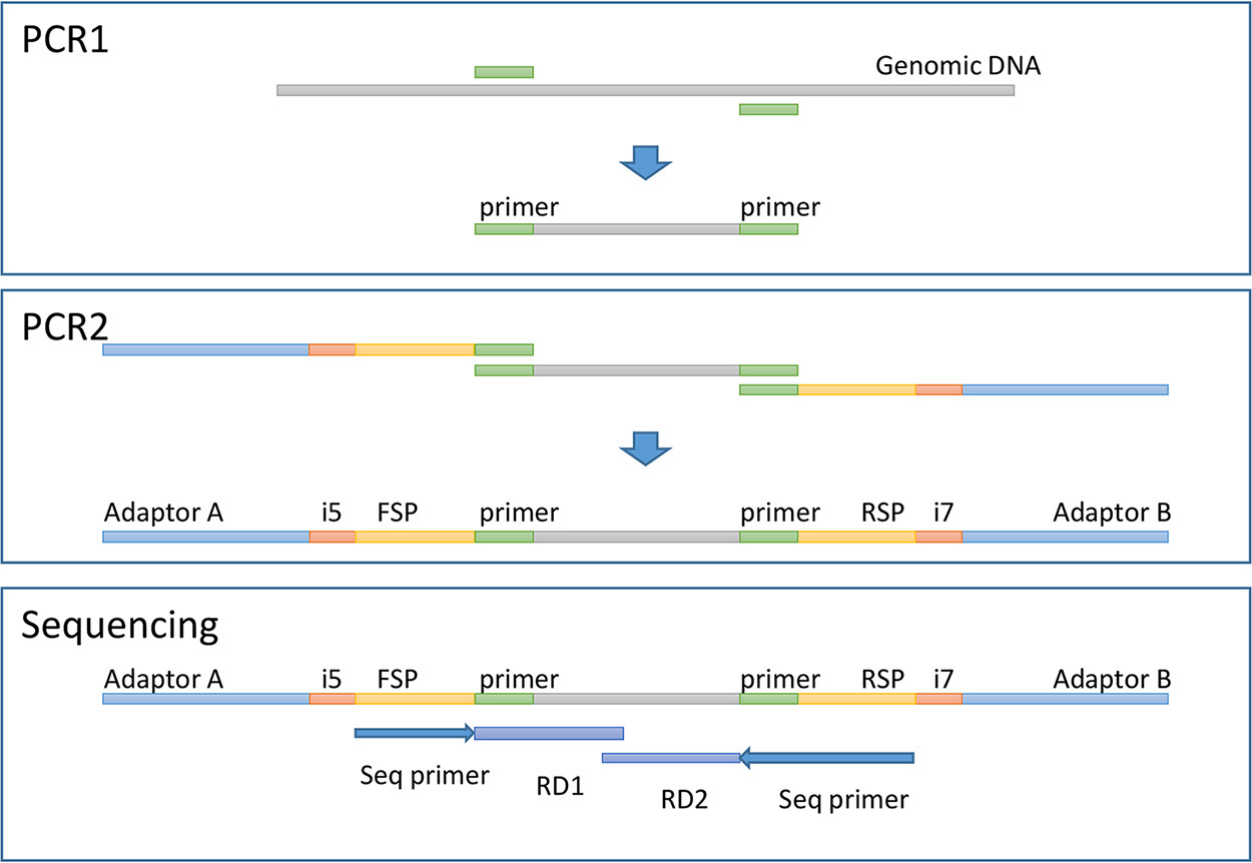
(Top) PCR1. Shown is a diagram of the PCR of the gene target area with genomic DNA as the template. Genomic template DNA was subjected to PCR-based amplification with one primer set targeting prokaryotes and three primer sets targeting eukaryotes. Each PCR was run in parallel. (Center) PCR2. Shown is a diagram of the attachment of the required elements to amplicons for MiSeq sequencing. The products from PCR1 were used as templates for the adaptor PCR, where adaptors, bar codes, and sequencing primer-binding sites were added. This was performed in parallel for each of the four primer sets. (Bottom) Sequencing. Shown is a diagram of the regions sequenced by MiSeq as RD1 and RD2. See the text for details.

For each primer pair, the rRNA gene was amplified using a short PCR setup as follows: initial denaturation at 95°C for 3 min was followed by 20 cycles of 95°C (30 s for 16S rRNA; 1 min for 18S rRNA), 60°C for 1 min, and 72°C for 30 s; final elongation was carried out at 72°C (7 min for 16S rRNA; 4 min for 18S rRNA). PCR was performed in a 25-μl volume, using the Extract-N-Amp PCR ReadyMix (Sigma-Aldrich, St. Louis, MO, USA) with 0.4 μM each primer and 2 μl of the template. This PCR is referred to as PCR1. The products from PCR1 were prepared for sequencing by a second PCR (PCR2) using the same PCR program. PCR2 attached an adaptor A, an index i5, and a forward sequencing primer site (FSP) to the 5′ ends of the amplicons and an adaptor B, an index i7, and a reverse sequencing primer site (RSP) to the 3′ ends of the amplicons (Fig. 1). Hence, four PCR products were generated for each sample. DNA was quantified using the Quant-IT high-sensitivity double-stranded DNA (dsDNA) assay kit (Thermo Fisher Scientific), and PCR2 products were pooled in equimolar amounts from the individual samples into the pooled amplicon library (PAL). Undesirable DNA amplicons were removed from the PAL by Agencourt AMPure XP bead (Beckman Coulter)-based purification in a two-step process. First, DNA fragments of <300 nucleotides (nt) were removed by use of 24 μl AMPure beads per 10 μl PAL, according to the manufacturer’s protocol, and were eluted in 40 μl Tris-EDTA (TE) buffer. Second, large DNA fragments of >1 kbp were removed by 16 μl AMPure beads per 10 μl AM1 (DNA resulting from the first AMPure bead treatment). The resulting AMPure bead-purified PAL was designated bPAL. The bPAL was diluted to its final concentration of 11.5 pM DNA with a 0.001 N NaOH concentration and was used for sequencing on the Illumina MiSeq desktop sequencer (Illumina Inc., San Diego, CA, USA). The library was sequenced with the 500-cycle MiSeq reagent kit, V2, in a 2 × 250-nt setup (Illumina Inc., San Diego, CA, USA).

Data analysis was performed using BION. The pipeline accepts raw sequences and includes steps for demultiplexing, primer extraction, sampling, sequence- and quality-based trimming and filtering, dereplication, clustering, chimera checking, identification of similarities to reference data, and taxonomic mapping and formatting. Nonoverlapping paired reads were allowed for analysis.

### Identification of *Acanthamoeba* genotypes.

BION automatically assigns a species name to *Acanthamoeba*-specific ribosomal DNA (rDNA) sequences. According to Martín-Pérez et al. (18), *Acanthamoeba* genotypes can be robustly grouped based on 18S rDNA sequences. Clustered FASTA files representing *Acanthamoeba-*specific DNA sequences were downloaded from the BION server and aligned with representative reference sequences downloaded from GenBank. The work by Martín-Pérez and colleagues (18) was used to inform the selection of reference sequences. The alignment was manually edited, and phylogenetic analysis of the edited alignment used the neighbor-joining algorithm as implemented in MEGA7 (19).

### Identification of organisms of potential clinical relevance.

A multitude of organisms have been reported to be involved in IK, including organisms commonly found on the skin (e.g., *Staphylococcus* and *Candida* species). Therefore, in the absence of a reference methodology, we reasoned that it would be useful, for a preliminary set of observations, to define a cutoff for the detection of organisms of potential clinical importance using a standardized proportion rather than a standardized absolute number of sequences, given the vast range in sequence read output (see Results).

Therefore, in *Acanthamoeba*-negative samples where ≥50% of the total output reflecting nonvertebrate DNA (i.e., nonhost DNA) could be assigned to a bacterial or fungal genus/species, this genus/species was considered to be of potential clinical relevance. This threshold reflected a conservative approach and was based on the rationale that if at least half of the sequences produced for any given sample could be attributed to a single microorganism, the likelihood that this organism was involved in the keratitis might be considered substantial.

### Ethical considerations.

This study complied with ASM's Ethical Guidelines (https://journals.asm.org/content/ethical-guidelines). Although the study involved DNA from human clinical samples (corneal scrapings), human DNA was not subjected to analysis. Moreover, the samples were anonymized prior to analysis by the 16S-18S assay. Hence, informed consent for publication was not deemed necessary, since patients could not be identified from any material in this article.

### Data availability.

*Acanthamoeba*-specific sequences produced by the NGS assay in this study have been submitted to GenBank under accession numbers MT919356 to MT919376.

## RESULTS

### 16S-18S sequence read output.

16S and 18S rRNA gene data were generated from all samples with a range of 3,909 to 381,110 reads per sample (median, 81,848 reads; interquartile range [IQR], 48,219 to 106,487 reads) (see Fig. S1 in the supplemental material). Verterbrate DNA (i.e., host DNA) represented a significant proportion of all sequences generated (median, 42,548 sequences; IQR, 24,861 to 64,661 sequences). Three to 74 genera were identified in each sample.

### Ability of the 16S-18S assay to identify *Acanthamoeba*-positive samples and usefulness of the sequences obtained for genotyping.

A total of 24 samples had been scored *Acanthamoeba* positive by real-time PCR; of these, 2 samples were considered weakly positive for *Acanthamoeba*. By the 16S-18S assay, *Acanthamoeba* rDNA was detected in 21 samples, all of which had been scored positive by real-time PCR (specificity, 100%). Meanwhile, the 16S-18S assay failed to detect *Acanthamoeba* DNA in three of the samples positive by real-time PCR, two of which were weakly positive (sensitivity, 88%). None of the samples identified as *Acanthamoeba* negative by real-time PCR were found positive by the 16S-18S assay.

For 16 samples, a result at the species level was returned by BION. The species identified were Acanthamoeba palestinensis (*n* = 2), Acanthamoeba hatchetti (*n* = 3), Acanthamoeba polyphaga (*n* = 5), Acanthamoeba castellani (*n* = 4), and Acanthamoeba mauritaniensis (*n* = 2). The remaining *Acanthamoeba* spp. (*n* = 5) could be identified only to the genus level. Based on phylogenetic analysis, a total of 19 sequences reflected genotype T4, and 2 sequences reflected genotype T6.

### Overall mapping of bacterial DNA in the samples.

The 16S rRNA gene sequences were analyzed at various taxonomic levels, ranging from phylum to species. The dominant phylum was *Proteobacteria*, which, on average, represented 67.2% of bacterial reads (range, 0 to 100%). *Firmicutes* (mean, 16%; range, 0 to 100%), *Actinobacteria* (mean, 10.3%; range, 0 to 72.1%), and *Bacteroidetes* (mean, 5.0%; range, 0 to 59.4%) were also highly represented. Other phyla, such as *Acidobacteria* (mean, <0.1%; range, 0 to 3.0%), *Fusobacteria* (mean, 0.6%; range, 0 to 21.4%), *Deinococcus*-*Thermus* (mean, 0.3%; range, 0 to 9.3%), *Chlamydiae* (mean, <0.1%; range, 0 to 76.2%), *Spirochaetes* (mean, <0.1%; range, 0 to 3.4%), *Tenericutes* (mean, <0.1%; range, 0 to 10.1%), *Thermatogae* (mean, <0.1%; range, 0 to 1.18%), and *Verrucomicrobia* (mean, <0.1; range, 0 to 2.8%), were present, but in smaller proportions. The remaining bacterial reads from a diverse set of phyla contributed very few reads (mean, 0.2%; range, 0 to 9.8%). The most common bacterial genera were *Pseudomonas*, Acinetobacter, *Propionibacterium*, and *Streptococcus*, but their relative proportions differed greatly between the samples.

### Organisms potentially causing nonviral IK detected in *Acanthamoeba*-negative samples.

In addition to *Acanthamoeba*, several other pathogens that could be involved in the development of IK were detected (Table 1). Fungal and bacterial species of potential clinical relevance were identified in 31 of the samples negative for *Acanthamoeba*; these included Pseudomonas aeruginosa (*n* = 11), *Moraxella* spp. (*n* = 6), Staphylococcus aureus (*n* = 2), *Fusarium* spp. (*n* = 4), and Candida albicans (*n* = 1). All these accounted for ≥50% of the total sequence output reflecting nonvertebrate DNA (i.e., nonhost DNA) in *Acanthamoeba*-negative samples.

**TABLE 1:** Organisms detected in *Acanthamoeba*-negative samples and reflecting at least 50% of sequence reads

Microbial genus/species^*a*^	No. of samples in which the organism was observed	Possible interpretation^*b*^
*Pseudomonas* spp.	12 (P. aeruginosa, 11; P. fragi, 1)	C
*Moraxella* spp.	6 (M. catarrhalis, 4; M. nonliquefaciens, 2)	C
Saccharomyces cerevisiae	5	S
*Fusarium* spp.	4	C
Malassezia globosa	4	S
*Streptococcus* spp.	4 (S. mitis, 2; S. dysgalactiae, 1; S. pneumoniae, 1)	C
Staphylococcus aureus	2	C
Candida albicans	1	C
Eikenella corrodens	1	C
Enterococcus faecium	1	C
Total	40	C (*n* = 31), S (*n* = 9)

a Listed according to frequency.

b S, more likely skin contamination; C, more likely clinical relevance.

## DISCUSSION

In this study, we examined the feasibility of the 16-18S assay as a diagnostic tool for the detection of *Acanthamoeba*-specific DNA in corneal scrapings received due to infectious keratitis (IK).

Our data demonstrate that the 16-18S assay was able to detect *Acanthamoeba*-specific DNA with a sensitivity of 88% and a specificity of 100% relative to *Acanthamoeba*-specific real-time PCR. As an added benefit, the *Acanthamoeba* sequences obtained by the 16S-18S assay served both to confirm the presence of *Acanthamoeba*-specific DNA and also enabled genotyping of the strains based on the sequence output analyzed by BION. Finally, we developed an automated algorithm that identified organisms represented by at least 50% of the total sequence read count in the sample as potentially clinically relevant nonviral causes of IK, and 31 (18%) of the *Acanthamoeba*-negative samples (*n* = 176) were positive for a clinically relevant bacterium or fungus.

Reports on NGS-based approaches to detecting and differentiating organisms involved in IK are still very limited. Li et al. applied metagenomics to a very limited set of paraffin-embedded samples (*n* = 16) (20). Prashanthi et al. used amplicon-based NGS of the internal transcribed spacer (ITS) region to characterize alterations in the ocular surface fungal microbiome in fungal keratitis (21). So far, however, no studies have been published on amplicon-based NGS of ribosomal rRNA genes from bacteria, fungi, and parasites in corneal scrapings.

The primers used in the 16S-18S assay have very limited specificity compared with the primers (and probe) used in the real-time PCR assay, and the fact that the 16S-18S assay was able to detect 21 out of 24 *Acanthamoeba*-positive samples is quite remarkable, especially considering the fact that 2 of the 24 samples scored positive by real-time PCR were categorized as “weakly positive,” so the 16S-18S assay identified 21 out of 22 clearly positive samples (95%). No other microorganism that could explain IK was found in these samples using the 16S-18S assay. Unfortunately, we did not have any clinical information on the samples, and it is not known to us whether the two faintly positive samples came from patients who had already been receiving *Acanthamoeba*-targeted treatment prior to sampling, which could explain the very weak signals. None of the three samples testing weakly positive by real-time PCR and negative by the 16S-18S assay were positive for other organisms relevant to IK by 16S-18S analysis; however, since data from local clinical microbiology laboratories were not available for analysis in the present study, we could not investigate whether these samples were from patients with IK potentially suffering from viral infections. The sensitivity of the real-time PCR used as a reference in the present study remains unknown. However, about 15% of all samples referred for *Acanthamoeba* PCR and tested by this real-time PCR in Denmark are positive. The ability to establish a diagnosis of AK correctly based on analysis of corneal scrapings relies not only on sufficiently sensitive microbiological methods but also on the ability to sample appropriately.

Establishing a diagnosis of AK is very difficult under normal circumstances, especially in the later stages of the disease, when very few trophozoites are present and the disease is dominated by cysts. It remains to be determined if the sensitivity of this analysis is high enough for routine diagnostics or whether it will remain a supplement to the specific RT-PCR. It is possible that the assay could prove useful in the initial stage of IK, when trophozoites are more abundant than they are in later stages.

Organisms from >75% of samples (16 of 21) were identified to the species level, providing useful information on the pathogens. Although it is encouraging that the microbiome platform enables species identification, this information should not stand alone but needs to be supplemented by genotype determination. Table 2 summarizes data from similar studies reflecting *Acanthamoeba* genotypes identified mainly in corneal scrapings, but also in corneal/nasal swabs, contact lenses, and contact lens solutions. As can be seen, T4 is the by far the most common genotype detected, accounting for >83% of the cases, followed in prevalence by T3, which accounts for 6% of the cases. As suggested by the distribution of data in Table 2, the data obtained in the present study add support to the claim that T4 is by far the most common genotype involved in human AK (22). Of note, we identified T6, a genotype rarely seen in AK, in two corneal scrapings. Walochnik and colleagues identified the T6 genotype when analyzing a “hypervirulent” strain of *Acanthamoeba* from a contact lens-wearing patient with keratitis whose case was managed in Austria (23). It remains unclear whether the genotypes involved in AK differ in terms of clinical course/severity and susceptibility to treatment. Given the rarity of the infection, multicenter prospective studies to investigate this question would be appropriate.

**TABLE 2:** Selection of studies showing the distribution of *Acanthamoeba* genotypes identified in *Acanthamoeba*-positive samples

Reference or source	Country	Sample material(s)	No. of samples analyzed	No. of samples with *Acanthamoeba* genotype:								
				T3	T4	T5	T6	T9	T10	T11	T13	T15
Behera et al. (24)	India	Cornea	18		16				2			
Rocha-Cabrera et al. (25)	Spain	Cornea	17		17							
Omaña-Molina et al. (26)	Mexico	Cornea	2	1	1							
Montalbano Di Filippo et al. (27)	Italy	Cornea (*n* = 12), lens solution (*n* = 19), swab (*n* = 24)	55	11	33					1		10
Arnalich-Montiel et al. (28)	Spain	Cornea	17	2	14					1		
Grün et al. (29)	Germany	Contact lens	1								1	
Cabello-Vilchez et al. (30)	Peru	Nasal swabs	21		20							1
Takaoka-Sughihara et al. (31)	Japan	Cornea	6		6							
Lorenzo-Morales et al. (32)	Spain	Cornea	1							1		
Abe and Kimata (33)	Japan	Cornea	7		7							
Sharifi et al. (34)	Sweden	Cornea (*n* = 10), contact lens (*n* = 1)	11	1	8					1		1
Zhao et al. (35)	China	Cornea	14		14							
Ledee et al. (36)	USA	Cornea	37		36	1						
Yera et al. (37)	France	Cornea	9	1	7					1		
Gatti et al. (38)	Italy	Cornea	15		15							
Hajialilo et al. (39)	Iran	Cornea	17		14			1		2		
Present study	Denmark	Cornea	21		19		2					
Total			269	16	227	1	2	1	2	7	1	12

In this study, we applied a strict criterion for determining which of the organisms detected could be considered potential contributors to the development of IK. Hence, only bacterial or fungal species that made up >50% of nonvertebrate sequence reads were included. Still, quite a few genera/species of potential clinical relevance were noticed. P. aeruginosa was detected in 11 samples and S. aureus in 2; both are well-known causes of IK. Several other bacterial species that could potentially be causes of IK were detected. Unsurprisingly, these bacteria were mainly bacterial commensals found in the upper airways and as part of the oropharyngeal environment, and the clinical significance of these findings should be evaluated on a case-to-case basis, just as it should be confirmed using conventional culturing. We did, however, adopt a very conservative algorithm to ensure that the bacterium identified was found at levels that warranted further clinical follow-up. This was also true for fungal species; we found four samples containing *Fusarium* spp. and one sample containing C. albicans, all of which are potential causes of keratitis and could be of clinical significance.

The availability and cost of this assay is also a concern that needs to be taken into account. In our setup, this assay requires 17.5 technician working hours and 6 molecular bioinformatics working hours. Downstream analysis of the NGS assay output has been automatized so that the freely available software BION annotates the sequence results automatically to taxonomic units, significantly reducing the amount of work related to sequence read analysis.

These findings are encouraging because they demonstrate the ability of the assay to detect and differentiate microorganisms that are usually found using conventional methods. Due to ethical considerations and limitations, this study could not include data from local clinical microbiology laboratories to confirm our findings and validate our algorithm. We suggest developing a prospective study where data from the 16S-18S assay can be compared with data from routine clinical microbiology analyses and other investigations used to establish a diagnosis of AK (such as confocal microscopy), since this will be crucial in determining the feasibility of this platform as a frontline screening tool for nonviral causes of IK. The microbiome platform is not dependent on viable bacteria or fungi in order to determine their presence, and it is conceivable that this analysis could provide new/additional information. It is not unusual for a patient to have been prescribed topical antibiotics prior to sampling for keratitis, and a diagnostic approach that is independent of the viability of pathogens could be of great value. Further studies comparing the 16S-18S platform to standard culturing in this setting would be of great value.

Since it is based on small-subunit (SSU) rRNA genes and not ITS regions, the 16S-18S assay does not have sufficient discriminatory power to identify molds to the species level, and subsequent analyses will be needed to make a full identification. The same issue pertains to *Streptococcus* spp., for which further testing will be needed for identification to the species level.

In conclusion, our study confirmed that the 16S-18S assay is able to detect the presence of *Acanthamoeba* spp. with a sensitivity of 88% and a specificity of 100%. The assay was able to provide valuable information on *Acanthamoeba* genotypes. Furthermore, the assay was able to detect bacterial and fungal pathogens potentially involved in IK; however, further studies are needed to ascertain the sensitivity of this analysis.

## Supplementary Material

Supplemental file 1
