# Supplementary material for: Detection and Identification of Acanthamoeba and Other Nonviral Causes of Infectious Keratitis in Corneal Scrapings by Real-Time PCR and Next-Generation Sequencing-Based 16S-18S Gene Analysis
Source: J Clin Microbiol. 2021 Jan 21;59(2):e02224-20. doi: 10.1128/JCM.02224-20 (PMC8111161; doi:10.1128/JCM.02224-20)
Supplement: Supplemental file 1 [file JCM.02224-20-s0001.pdf]

Supplementary Figure 1A

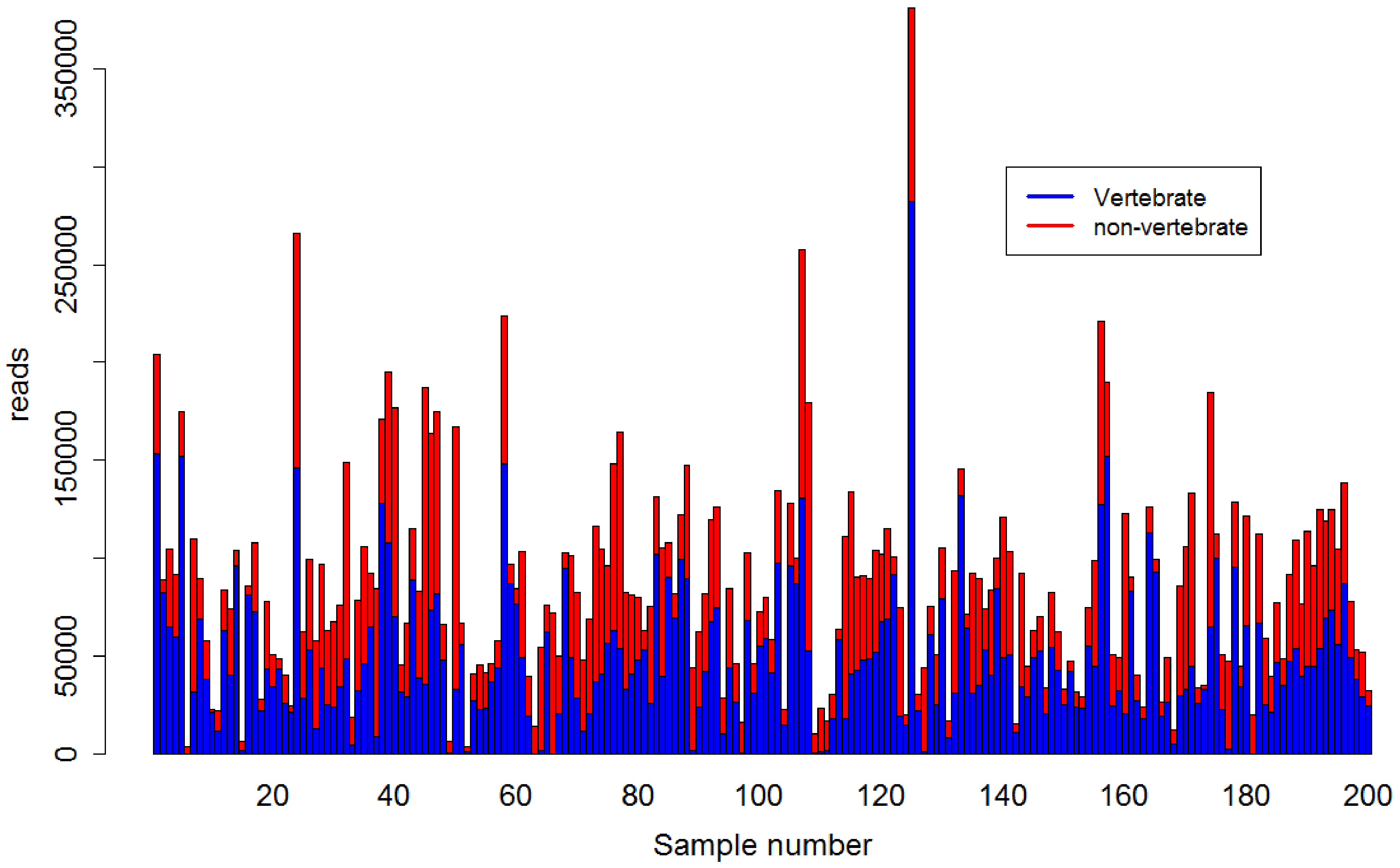

Supplementary Figure 1B

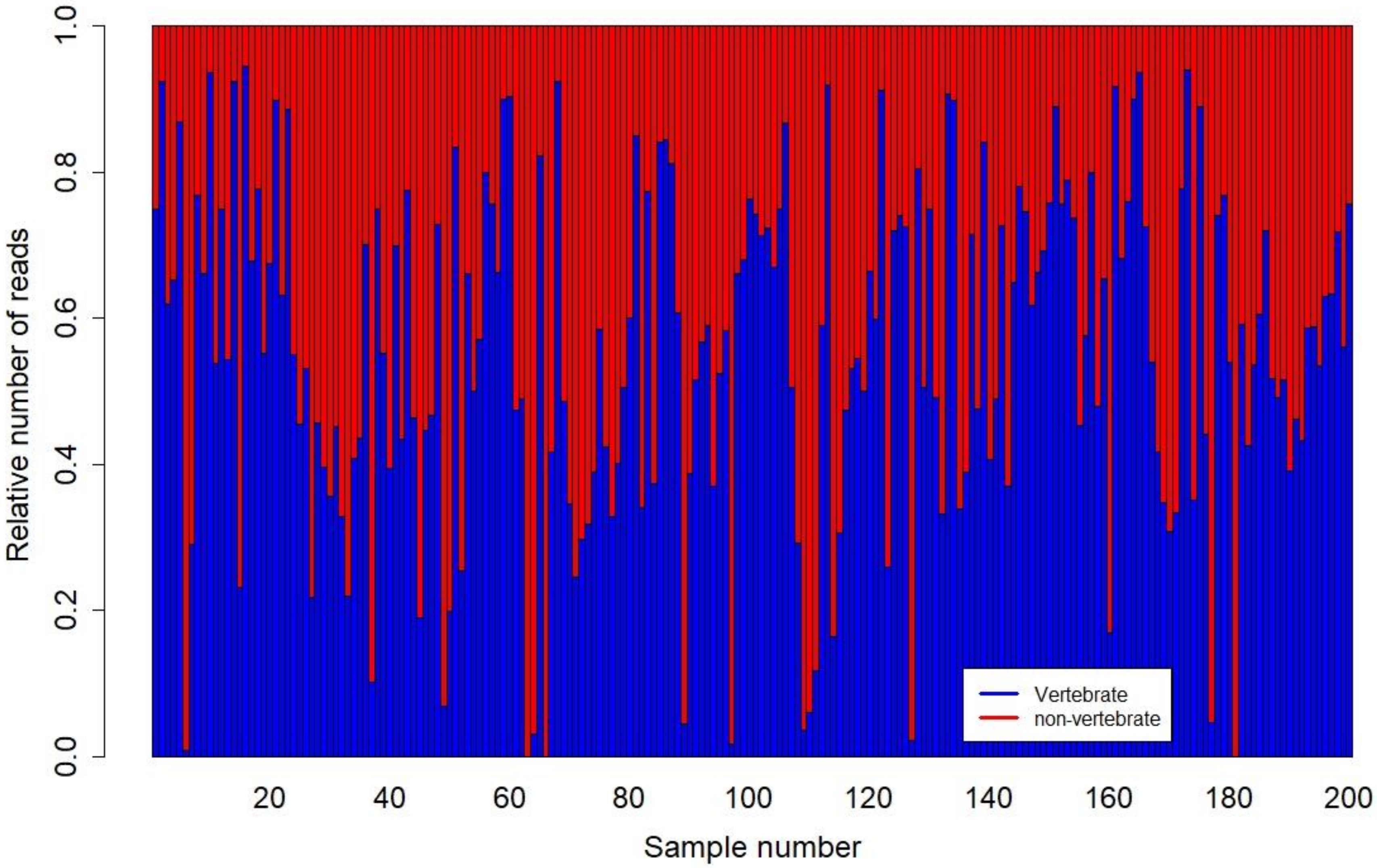

Supplementary Figure 1C

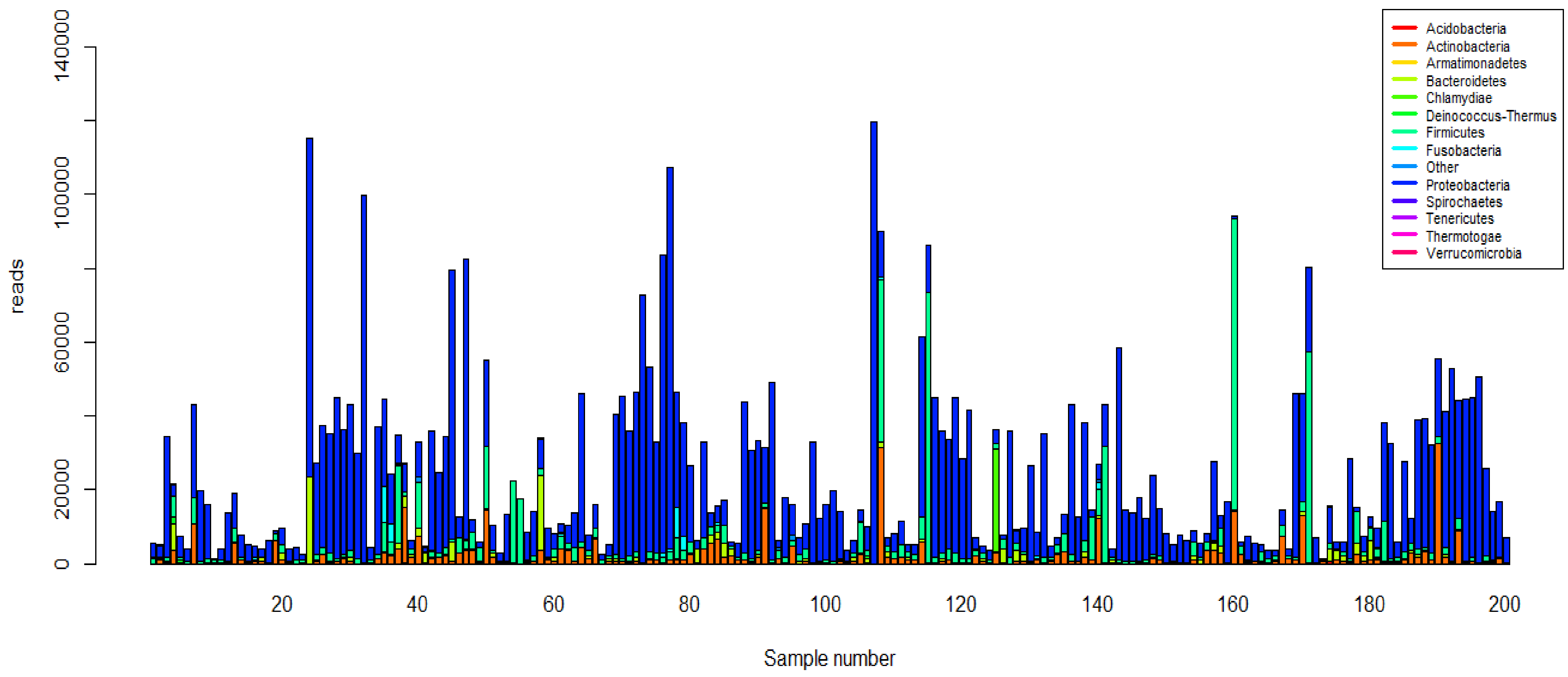

Supplementary Figure 1. A: Total number of reads attributable to vertebrates and non-vertebrates. B: Relative number of reads attributable to vertebrates and non-vertebrates. C: Number of bacterial reads attributable to different genera.
